# A novel simulator-based checklist for evaluating residents' competence in cerebral angiography in China

**DOI:** 10.3389/fneur.2023.1122257

**Published:** 2023-02-16

**Authors:** Xuxia Yi, Gang Wang, Nai Zhang, Weixin Si, Jianping Lv

**Affiliations:** ^1^Department of Neurosurgery, Guangzhou First People's Hospital, Guangzhou, China; ^2^National Center for Mental Health, China, Beijing, China; ^3^Department of Neurosurgery, Tianjin Medical University General Hospital, Tianjin, China; ^4^Shenzhen Institute of Advanced Technology, Chinese Academy of Sciences, Shenzhen, China

**Keywords:** checklist, reliability, GRS of endovascular performance, cerebral angiography, simulation

## Abstract

**Background:**

Nowadays, with the fast-increasing demand for neuro-endovascular therapy, surgeons in this field are in urgent need. Unfortunately, there is still no formal skill assessment in neuro-endovascular therapy in China.

**Methods:**

We used a Delphi method to design a newly objective checklist for standards of cerebrovascular angiography in China and evaluated its validity and reliability. A total of 19 neuro-residents with no interventional experience and 19 neuro-endovascular surgeons from two centers (Guangzhou and Tianjin) were recruited; they were divided into two groups: residents and surgeons. Residents completed a simulation-based cerebrovascular angiography operation training before assessment. Assessments were under live and video record forms with two tools: the existing global rating scale (GRS) of endovascular performance and the new checklist.

**Results:**

The average scores of residents were significantly increased after training in two centers (*p* < 0.05). There is good consistency between GRS and the checklist (*p* = 0.856). Intra-rater reliability (Spearman's rho) of the checklist was >0.9, and the same result was also observed in raters between different centers and different assessment forms (*p* < 0.001, rho > 0.9). The reliability of the checklist was higher than that of the GRS (Kendall's harmonious coefficient is 0.849, while GRS is 0.684).

**Conclusion:**

The newly developed checklist appears reliable and valid for evaluating the technical performance of cerebral angiography and differentiating between trained and untrained trainees' performance well. For its efficiency, our method has been proven to be a feasible tool for resident angiography examination in certification nationwide.

## 1. Introduction

In most vascular surgery centers, endovascular treatments have now superseded open surgical repair in the management of a large number of pathologies (1). Neuro-interventional surgery has been carried out in more than 700 hospitals in China, nearly 3,500 physicians were engaged in this field by 2018, and 124,100 operations were performed nationwide in 2019 (2). The increase in therapeutic endovascular treatment options has also led to a need for training in endovascular skills for the practitioners of the future. With the development of standardized residency training in China since 2014, most of the fresh interventional surgeons were trained under a residency program. Limited by several factors, qualification evaluation for neuro-interventional surgeons still remains like written mold, although government authorities in China demand that doctors who prepare to be neuro-interventional surgeons be trained and qualified (3).

These factors include the uneven distribution and development of medical resources and competencies of physicians across different regions in China (4), work-hour restrictions and changes in medicine (5), and the complexity of clinical assessment, involved ethics and fairness. These traditional examination methods only assess basic theoretical knowledge and do not evaluate technical performance (6), which cannot ensure a licensed and certified surgeon is capable of delivering quality care. In addition to the above difficult situation, the assessment of methodical problems and a blank assessment tool also block the implementation of the qualifying policy.

Simulation-based assessment (SBA) is a great idea to solve assessment methodical problems, which has been incorporated in a number of high-stakes certification and licensure examinations (7–9). At present, many intervention centers or simulation centers have developed their assessment tools (9–12) for training and assessment or even certification and licensure examinations. The two most commonly used are checklists and GRS, and the most widely used is the GRS of endovascular performance, which is adapted from a previously validated scoring system (13). There were two common limitations among these assessment tools, which are as follows: (1) not unique to diagnostic neurovascular intervention, they can be applied to all interventional procedures; and (2) rater-dependent, GRS is subjective, which allows a rater to evaluate the degree (on a 1 to 5 scale) of a learner performing all steps in a given assessment exercise (13). A Likert rating scale (0–4; 4 being best) was used for checklist grading (9), which means they were semi-subjective. Another issue is that the objective is to assess the examinees' competence to complete the surgery while ignoring the standardization of basic surgical steps.

As mentioned earlier, the development of neuro-interventional training in different centers has obvious regional differences since regional representative hospitals have their training protocols and assessment methods. There is no standard method existing for skill evaluation. In the face of important assessments such as the national examination for readiness and competence for certification, it is difficult to guarantee the fairness of the results. Moreover, large-scale examiner training needs to consume a lot of time, manpower, and economic costs. These existing assessment tools are difficult to implement for such an important examination in China. To overcome subjectivity, an assessment tool should be designed so that there is little room for assessor interpretation, such as with a uniform rubric.

Therefore, with the support of the Chinese National Medical Examination Center, we conducted the following research. The newly designed objective checklist should reflect and distinguish the different levers of examinees' competency reliably. Different from the existing GRS of endovascular performance and checklists, the evaluation indicators of the scale are combined with the objective automatic metrics of the simulator, which means the checklist is more objective and has less rater bias. First, the score-based decisions must be validated and demonstrated to be reliable before using this scale to evaluate the ability of the examinee.

## 2. Materials and methods

This is a multi-stepped approach with mixed methods to assess the validity, reliability, and applicability of our new objective checklist for cerebrovascular angiography. The study was approved by the Committee of Guangzhou First People's Hospital, Guangzhou, China.

### 2.1. Development of the assessment tool

The checklist was developed by a team of nine experts from three hospitals: six key members of the Chinese Society for Neurointervention, with over 2,000 neuro-endovascular cases' experiences, two persons with experience in both interventional and open surgery fields of at least 20 years, and one expert in measurement.

We began with the literature “Chinese expert Consensus on the Operation Specification of Cerebrovascular Angiography” (14) to select initial 40 items of surgical parameters in the main procedures of cerebrovascular angiography with the addition of automatic metrics from virtual reality simulator (total fluoroscope time, total time, amount of contrast used, and handling events), which can adequately help interventionalists in grading intervention difficulty (15). Performance metrics used a Delphi method through three rounds of survey (16) to develop the consensus opinion on kept/omitted items, appropriateness of each item's content, and weight of each item. The first two rounds were *via* a video conference, and the last was conducted through offline discussion. The tool was reviewed for its completeness, relevance, and representativeness and normalized (17). The final form (maximum score = 100) included the following three domains: part 1: Prepare (five items, max score 8); part 2: Steps of the Procedure (13 items, max score 82); and part 3: Diagnosis (two items, max score 10). The final form is shown in Supplementary Table 1. We based specific detailed descriptions of angiography steps with emphasis on the entirety of the procedure, including checking patient identity, asepsis, handling catheter and wire, the capture of the standard picture, correct use of contrast, and reading angiogram.

### 2.2. Assessment procedure

A total of 38 vascular residents and neurosurgeons from the Department of Neurosurgery, Guangzhou First People's Hospital, and Tianjin Medical University General Hospital were recruited for this research. The trainees were divided into two groups: residents (residents with no endovascular experience) and surgeons (neurosurgeons with endovascular experience of 100–200 cases). The residents received a 2-day (3 h) endovascular skill training course, including didactic teaching and diagnostic cerebral angiography operation training on simulator Mentice Vascular Intervention Simulation Trainer (VIST; Gothenburg, Sweden, Guangzhou center) or Simbionix ANGIO Mentor (Simbionix, Cleveland, OH, Tianjin center).

Before training, a didactic course, including instruction on catheter handling, device selection, endovascular techniques, and manipulating simulators, was given to residents. The training time of all residents was 3 h. One-on-one training was provided throughout the training sessions by senior neuro-interventional doctors. Performance on cases with aortic arch type I (to avoid case difficulty bias) was used to assess early on the 1st day after didactic teaching, and this was repeated after the training by two highly experienced surgeons (mean experience of 20 years). Surgeons were also tested after learning to manipulate the simulator for 30 min.

The evaluation forms were used by on-site observation and video (screen recording) alike with two assessment tools: the checklist and the GRS of endovascular performance (Supplementary Tables 1, 2). Each examinee was evaluated by two raters (a total of four from two hospitals with a mean of 20 years of experience). The raters were trained by lecture and literally before the assessment. Screen recording recorded the overall core part of the procedure (Steps of the Procedure) to ensure an overview of the entire critical performance sequence; then, they were scored by all raters from two centers using the two tools. Video scoring was reviewed a month after direct observation (18, 19). The order of videos was randomized, so the raters were blinded to the identity of operators.

### 2.3. Statistical analysis

All data were imported into SPSS 21.0 for analysis. Evidence of validity was provided by comparing scores between different groups with different clinic experiences in two centers. Data of DI for the checklist were evaluated aspect of construct validity. Another piece of evidence was the relationship between the total scores obtained from the two assessment tools. The “inter-rater” and “intra-rater” reliability were tested with Spearman's rho tests. The internal consistency of the checklist was evaluated by assessing the value of Kendall's harmonious coefficient, including different assessment forms, centers, and tools. The significance level in all hypothesis testing procedures was predetermined at *p* = 0.05.

## 3. Results

The basic background information such as age, sex, clinical experience, and angiography cases is listed in Table 1, and the two cohorts were identical in terms of background, except for angiography cases.

**Table 1:** Background information of participants.

	**Residents**			**Surgeons**		
	**Guangzhou**	**Tianjin**	**Person correlation coefficient (** ***p*** **-value)**	**Guangzhou**	**Tianjin**	**Person correlation coefficient (** ***p-*** **value)**
M	8	10		9	10	
F	1	0		0	0	
Age (mean)	26	26.2		30.1	35.3	
Clinical working years (mean)	2.8	0.6	0.329	3.6	9.5	0.444
Experience with vascular/year (mean)m	0	0	/	158	275	0.050

M, male; f, female.

Average scores across each group at every time are shown in Table 2. The scores in the surgeons group in Tianjin were significantly higher than any other cohort (*p* < 0.05), which positively correlated with the number of angiography experiences (Table 1).

**Table 2:** Comparison of mean checklist scores across each group between the two centers.

	**Guangzhou**				**Tianjin**			
	**Pre-training**	**Post-training**	**Surgeon**	**Sig**	**Pre-training**	**Post-training**	**Surgeon**	**Sig**
Prepare	5.6	7.9	6.3	0.051	4.2	7.6	7	0.003
Procedure	41.6	56.1	63.7	0.004	41	59	72.5	0.002
Diagnosis	1.6	8.4	7.3	0.013	1.1	6.2	9.2.	< 0.001
Total Score	48.8	72.4	77.3	0.000	46.3	72.8	88.9	< 0.001

**Figure 1:**
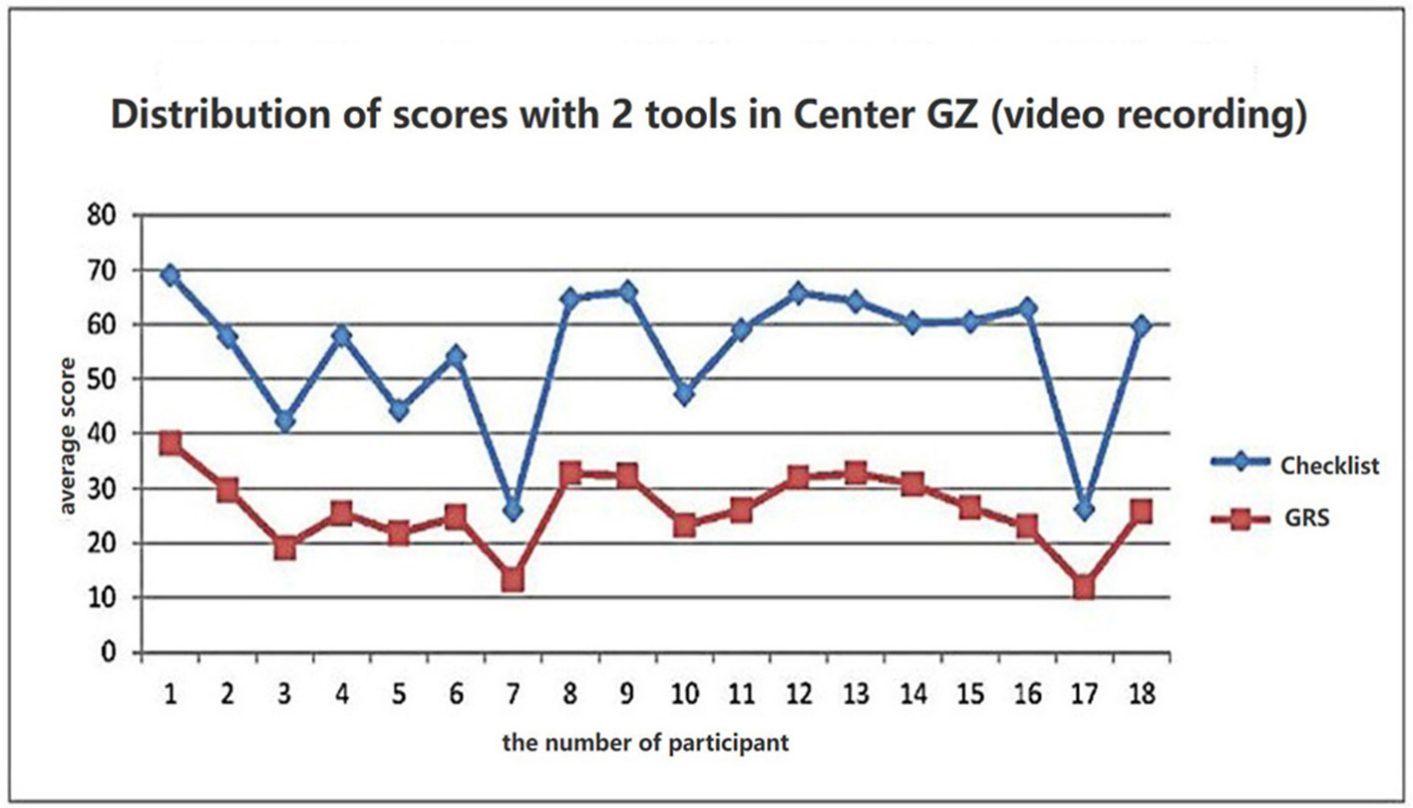
Mean score distribution of the resident group with GRS and checklist. Score differentiation between the two assessment tools, Pearson's correlation 0.9.

The scores were placed based on average scores per item in Table 3. The average discrimination index (DI) and difficulty coefficient (DC) of each item were also calculated. DI was more than 0.6 indicating good homogeneity. Based on data presented in Table 4, the total scores of the raters using the two tools, with both assessment forms, were gratifying reliability indices (Cronbach's α more than 0.6). The comparison with the score sheet showed that the score across the two evaluation tools had a significant positive correlation (Figure 1), indicating that the checklist tool is consistent with the GRS internationally approved.

**Table 3:** Checklist item performance averages of difficulty coefficient and differentiation index for all participants.

**Items (total score)**	**Mean score**	**SD**	**Difficulty coefficient (DC)**	**Differentiation index (DI)**
1 (1)	0.8	0.36	0.71	0.57
2 (1)	0.8	0.39	0.64	0.71
3 (2)	1.8	0.50	0.76	0.43
4 (3)	1.7	0.52	0.75	0.5
5 (3)	1.5	0.64	0.68	0.64
6 (6)	5.6	1.27	0.88	0.88
7 (5)	4.4	1.39	0.63	0.45
8 (6)	4.0	2.13	0.6	0.81
9 (5)	4.3	1.19	0.77	0.46
10 (6)	4.3	1.10	0.69	0.33
11 (6)	5.1	1.65	0.76	0.48
12 (6)	5.0	1.60	0.75	0.50
13 (6)	4.7	1.89	0.68	0.64
14 (6)	4.7	1.68	0.7	0.60
15 (6)	4.9	0.75	0.76	0.48
16 (12)	7.2	2.61	0.51	0.97
17 (8)	7.3	1.17	0.71	0.57
18 (5)	3.1	1.55	0.36	0.71
19 (5)	3.5	2.22	0.5	1
20 (5)	3.1	1.83	0.5	0.89

**Table 4:** Rater reliability (Cronbach's α coefficient) of on-site observation and video recording-based scores by GRS and checklist.

**Raters**	**On-stie observation**		**Video recording**	
	**GRS**	**Checklist**	**GRS**	**Checklist**
Rater 1	0.721	0.895	0.85	0.893
Rater 2	0.705	0.893	0.831	0.893
Rater 3	0.653	0.829	0.661	0.849
Rater 4	0.694	0.821	0.793	0.979

According to data presented in Table 5, for each rater, there was a strong positive statistically significant intra-rater reliability between the two evaluation forms (for all raters, *rho* > 0.9, *p* < 0.001). The same result was also observed in raters between different centers (*p* < 0.001, *rho* > 0.9).

**Table 5:** Reliability for the mean score on different centers, intra-rater, and different tools for the same examinee inter-hospitals.

	**Spearman's rho**	***p-*Value**
**1. Centers**		
Guangzhou (checklist)	0.975	< 0.001
Tianjin (checklist)	0.930	< 0.001
**2. Intra-rater**		
Rater 1	0.974	< 0.001
Rater 2	0.933	0.053
Rater 3	0.961	< 0.001
Rater 4	0.929	< 0.001
**3. Tools**		
GRS	0.948	< 0.001
Checklis	0.61	0.061

The vast majority of the scores of the examiners using the two tools for both periods of the assessment showed that the checklist tool was more satisfactory for intra- or extra-reliability indices than GRS (Table 6; Kendall's harmonious coefficient is 0.849, while GRS is 0.684).

**Table 6:** Reliability results of the two tools used by the four raters.

	**Kendall's harmonious coefficient**	***p-*Value**
GRS	0.684	0.001
Checklist	0.849	< 0.001

## 4. Discussion

In this study, we designed and implemented a new objective checklist to assess the residents' technical ability to operate the standard procedure of cerebral angiography, for it is the basic skill of cerebrovascular intervention. This is the first assessment tool designed based on Chinese expert consensus for Chinese trainees. The assessment is based on VR simulators, which can be performed in all steps of intervention except for arterial puncture. This is because this procedure is complex and better with another assessment tool. For a simulation module to be considered valid, it should ideally be assessed for validity, and the contemporary meaning of validity is a unitary concept with multiple aspects that considers construct validity as the whole of validity (20).

Validity evidence refers to data collected to assign a meaningful interpretation of assessment scores (21). The checklist was developed stepwise by a Delphi method with nine experts, which could ensure quality content validity. Unfortunately, a rate of agreement (RoA) as a measure of consensus among the experts was not calculated in this study, which is an appropriate measure of consensus particularly when Likert scales are used (22).

The data from this study support the construct validity of the assessment scale because the scale was able to reflect the increased competence of residents, to differentiate with and without experienced trainees, or with different cases experience (*p* < 0.05). The study showed a significant improvement in all domains of residents' skills, but mainly in the steps of the procedure and then diagnosis. The detailed nature of the checklist makes it an interesting tool for training purposes or surgical plans with complicated cases, to find out the obstacle steps.

After training, there was no significant difference between experienced and non-experienced trainees in GZ, which means group residents take a shorter time to achieve the same scores compared with the surgeons group. A study by the Aggarwal team (23) showed that there is an expected learning curve in performing simulated endovascular tasks. The results also suggested that no correlation exists between an individual's operative experience as reported by case logs and their technical performance (24). Another explanation is that cerebral angiography is a basic skill for intervention, and trainees can grasp it in a short time with standard training. While there was a different result between the residents and surgeons in TJ, it may be due to the surgeons having more angiography experience. The inconsistent results indicated that the volume of operations should not be a reliable and direct measure of technical skill (25). Of course, it will be more comprehensive if the learning curve was measured by patient outcomes (morbidity or mortality) or by procedure-specific metrics (blood loss and operative time) (26). To judge the score of the checklist can reflect the achievement of the examinee, we need to evaluate the performance of residents with the checklist in the operative room (OR) compared to simulations in the future.

As can be seen from the difference in scores before and after the training, the scores of the residents group in key items 3, 5, and 10 increased significantly after training, which affected the total score. The variation trends of 11 and 12 were consistent with the total score. The checklist not only helps identify which trainees have not yet achieved competence but also reveals specific areas of angiography in which a trainee needs further reinforcement and experience.

The scores of the experienced group were also different, especially because the score was positively correlated with the number of angiography cases, which was consistent with actual clinical experience. Meanwhile, it is combined with automatic metrics on the simulator, including “catheter scraping vessel wall” and “catheter moving without support of wire,” to avoid subjective deviation or omission of examiners in key steps.

The data of correlation with GRS and Cronbach's α suggest that the checklist is not redundant, and the latter could be a useful tool in the assessment of surgical competence. Cronbach's α and Spearman's rho of GRS were both lower than that of the checklist. Similar results could be seen in other research (27, 28). Sarker et al. (29) used error-based checklists to evaluate the performance of the senior surgeons and showed high inter-rater reliability (*k* value 0.79–0.84; *p* < 0.05). Procedure-based assessments (6) also possessed high inter-rater reliability (*G* > 0.8, using three assessors for the same index procedure) but were very procedure-specific and long (checklist of up to 62 items), which limited its practicality for use in evaluating common endovascular procedures.

With the gradually increasing introduction of newly developed tools for the assessment of technical skills into practice, it is important to define the role of examiners as well (30). To minimize rater bias, all raters in this study were experts in neurovascular disease. The scores of the new objective checklist showed a strong positive intra-rater reliability than GRS (*rho* >0.9). The geographic variation, non-uniform standards, and different medical education backgrounds of raters cannot be ignored after all. There was no specific and clear criterion in every item for GRS, raters exhibited more random variability than with the checklist (31). The positive results indicating the new assessment tool may be used to measure angiographic skills on the simulator with a rater bias-free. Its superiority needs to be validated by a different level of raters, and limited experts cannot meet a large number of examinees after all.

Discussions have arisen in the past as to whether one should rate surgical performance in an on-site or video-based manner (32–34). On-site assessment enables all aspects of the procedure to be rated, and video-based assessment enables the rater to view the videos at their convenience, which has led to the increased popularity of blinded video assessment of surgical skills (33). In this study, screen recording showed the same results as on live, indicating that consistency can be guaranteed even if the same rater is reassessed over a period of time. The objective checklist could minimize the relative influence of subjection on-site or incompleteness under video recording. Another detail that should be noticed is that three residents did not get increased scores at live observation because of nervousness, and this situation did not happen during the video recording. Video recording should be improved to record the whole scene when the examinee operates beginning with Prepare.

There is no doubt as to the value of surgical skills assessment, not only for evaluating training effect and qualification but also for estimating case difficulty in the clinic. The purpose of our study is to provide the examiner with a standardized tool in licensure and certification SBA to assure the individual who passes the examination can also adapt to practice. Following the above-mentioned checklist design did bring almost negligible subjective rater bias, repeating the study with multiple centers involved may provide more significant results.

It is worth noting that the data mentioned earlier only verified to reliability and validity of the checklist. In the future, to apply for certification examination, we must need more samples of residents from different centers to determine the passing line forward. Furthermore, we have to evaluate the reliability of diverse raters, such as peers (those of the same career tract) or junior faculty, to ensure enough competent raters to meet the large examinations. In this regard, a novel and step-by-step study need to be performed.

## 5. Conclusion

In this study, we designed an objective assessment tool for the cerebrovascular angiography performance of residents/trainees. After that, we made a preliminary feasibility study, and the results indicated three issues: first, the score can be differentiated between trained and untrained trainees, while our proposed tool also positively correlates with the GRS. Second, the checklist assesses the delicate step of performance. Third, the new assessment tool was feasible and acceptable to both examiner and faculty with valuable high consistency.

## Data availability statement

The original contributions presented in the study are included in the article/Supplementary material, further inquiries can be directed to the corresponding author.

## Ethics statement

The studies involving human participants were reviewed and approved by Guangzhou First People's Hospital, Guangzhou, China. Written informed consent from the participants or participants' legal guardian/next of kin was not required to participate in this study in accordance with the national legislation and the institutional requirements.

## Author contributions

JL concepted and supervised the study. XY, NZ, and GW carried out the study. XY analyzed, interpreted the data, and drafted the manuscript. WS critically revised the manuscript for important intellectual content. All authors contributed to the article and approved the submitted version.
