# Supplementary material for: A novel simulator-based checklist for evaluating residents' competence in cerebral angiography in China
Source: Front Neurol. 2023 Feb 16;14:1122257. doi: 10.3389/fneur.2023.1122257 (PMC9978472; doi:10.3389/fneur.2023.1122257)
Supplement: Supplementary file 1 [file Table_1.DOCX]

Supplementary Material

A Novel Simulator-based Checklist for Evaluating Residents’ Competence in Cerebral Angiography in China

Xuxia Yi, Weixin Si, Gang Wang, Nai Zhang and Jianping Lv^*^

*** Correspondence:** Jianping Lv: ljpwhx@163.com

# Supplementary Figures and Tables

**TABLE 1** Checklist for Diagnostic Angiography

| Name: Done correctly score  (total: 100) | | |
| --- | --- | --- |
| Prepare |  |  |
| 1. IF check patient’s identity | 1 |  |
| 2. IF check patient’s laboratory test results  3. IF sign informed consent  4. IF keep aseptic along the procedure | 1  2  2 |  |
| 5. IF skin preparation and draping completed | 2 |  |
| Procedure |  |  |
| 1. Choose the correct guide wire and catheter  2. Use Y valve and high-pressure drip  3. Able to walk catheter back over the wire(simulator alarm:selective/catheter moving without support of wire) | 6  5  6 |  |
| 4. Conduct Aortic arch angiography: (1) Accurate positioning of catheter tip, (2) correct projection Angle, (3) complete display of branch vessels, correct contrast agent dosage and speed (4) contrast dose of 30ml, (5) speed not more than 15mL /s) | 5 |  |
| 5.Conduct right common carotid artery angiography: (1) Accurate positioning of catheter tip, (2) correct projection Angle, (3) complete display of branch vessels, correct contrast agent dosage and speed (4) contrast dose of 30ml, (5) speed not more than 15mL /s) | 6 |  |
| 6.Conduct left common carotid artery angiography: (1) Accurate positioning of catheter tip, (2) correct projection Angle, (3) complete display of branch vessels, correct contrast agent dosage and speed (4) contrast dose of 30ml, (5) speed not more than 15mL /s) | 6 |  |
| 7. Conduct left subclavian artery angiography: (1) Accurate positioning of catheter tip, (2) correct projection Angle, (3) complete display of branch vessels, correct contrast agent dosage and speed (4) contrast dose of 30ml, (5) speed not more than 15mL /s) | 6 |  |
| 8. Conduct right subclavian artery angiography: (1) Accurate positioning of catheter tip, (2) correct projection Angle, (3) complete display of branch vessels, correct contrast agent dosage and speed (4) contrast dose of 30ml, (5) speed not more than 15mL /s) | 6 |  |
| 9.Keep guidewire catheter in the vessel overtime (simulator alam: selective/catheter scraping vessel wall) | 5 |  |
| 10.Total exposure time (< 12 points for 10 minutes, 9 points for 10-15 minutes, 6 points for 15-20 minutes, 3 points for >20 minutes) | 12 |  |
| 11. Able to position tip of catheter at top or bottom of screen and center image | 8 |  |
| 12. Total operation time (<15 minutes = 6 points, 15-20 minutes = 4 points, 20-25 = 2 points, >25 = 1 point) | 6 |  |
| 13. Notice the vital signs during procedure（able to answer the right numerical value on the monitor | 5 |  |
| Diagnosis |  |  |
| 1. Diagnosis pathology the correctly | 5 |  |
| 1. Able to measure accurately | 5 |  |

**TABLE 2** Global Rating Scale of Endovascular Performance

| 1 | 2 | 3 | 4 | 5 |
| --- | --- | --- | --- | --- |
| 1.Time and motion Many unnecessary moves |  | Efficient time/motion; some unnecessary moves |  | Clear economy of motion; maximum efficiency |
| 2.Wire and catheter handling Repeatedly makes awkward, tentative moves; inappropriate use |  | Competent use; occasionally stiff or awkward |  | Fluid moves; no awkwardness |
| 3.Awareness of wire position Seldom aware of wire position |  | Mostly aware; occasionally unaware of position |  | Always aware of wire position |
| 4.Maintenance of wire stability Rarely maintains wire stability; loses wire access |  | Wire usually stable; occasionally forward/backward motion |  | Wire always stable; no loss of wire access |
| 5.Awareness of fluoroscopy usage Excessive use of fluoro |  | Appropriate use; some unnecessary use |  | Clear economy of fluoro; maximum efficiency |
| 6.Precision of wire/catheter technique Imprecise technique; frequent overshooting |  | Precise technique; occasional overshooting |  | Perfect precise technique |
| 7.Flow of operation Frequently stopped; seemed unaware of next move |  | Some forward planning; reasonable progression of procedure |  | Obviously planned course; effortless flow |
| 8.Knowledge of procedure Knew all important steps of procedure Deficient knowledge |  | Knew all important steps of procedure |  | Familiar with all aspects of procedure |
| 9.Quality of final product Very poor |  | Acceptable |  | Clearly superior |
| 10.Ability to complete the case Not able to complete case |  | Able to complete case with assistance |  | Able to complete case independently |
| 11.Need for verbal prompts Repeatedly needed prompts | 2 | Needed prompts sometimes | 4 | Able to complete the case without prompts |
| 12.Attending takeover Occurred at every stage |  | Occurred during some portions of the procedure |  | Able to complete the case without attending takeover |
